# Factors associated with inflammation in preschool children and women of reproductive age: Biomarkers Reflecting Inflammation and Nutritional Determinants of Anemia (BRINDA) project

**DOI:** 10.3945/ajcn.116.142315

**Published:** 2017-06-14

**Authors:** Rebecca D Merrill, Rachel M Burke, Christine A Northrop-Clewes, Pura Rayco-Solon, Rafael Flores-Ayala, Sorrel ML Namaste, Mary K Serdula, Parminder S Suchdev

**Affiliations:** 1 Nutrition Branch, CDC, Atlanta, GA;; 2 Emory University, Rollins School of Public Health, Atlanta, GA;; 3 Independent Public Health Consultant, Cambridge, United Kingdom;; 4 Department of Nutrition for Health and Development, WHO, Geneva, Switzerland;; 5 Strengthening Partnerships, Results, and Innovations in Nutrition Globally, Arlington, VA; and; 6 Helen Keller International, Washington, DC

**Keywords:** acute-phase response, inflammation, obesity, stunting, women and children

## Abstract

**Background:** In many settings, populations experience recurrent exposure to inflammatory agents that catalyze fluctuations in the concentrations of acute-phase proteins and certain micronutrient biomarkers such as C-reactive protein (CRP), α-1-acid glycoprotein (AGP), ferritin, and retinol. Few data are available on the prevalence and predictors of inflammation in diverse settings.

**Objective:** We aimed to assess the relation between inflammation (CRP concentration >5 mg/L or AGP concentration >1 g/L) and covariates, such as demographics, reported illness, and anthropometric status, in preschool children (PSC) (age range: 6–59 mo) and women of reproductive age (WRA) (age range: 15–49 y).

**Design:** Cross-sectional data from the Biomarkers Reflecting Inflammation and Nutritional Determinants of Anemia (BRINDA) project from 29,765 PSC in 16 surveys and 25,731 WRA in 10 surveys were used to model bivariable and multivariable relations.

**Results:** The inflammation prevalence was 6.0–40.2% in PSC and 7.9–29.5% in WRA (elevated CRP) and 21.2–64.3% in PSC and 7.1–26.7% in WRA (elevated AGP). In PSC, inflammation was consistently positively associated with recent fever and malaria but not with other recent illnesses. In multivariable models that were adjusted for age, sex, urban or rural residence, and socioeconomic status, elevated AGP was positively associated with stunting (height-for-age *z* score <−2) in 7 of 10 surveys. In WRA, elevated CRP was positively associated with obesity [body mass index (in kg/m^2^) ≥30] in 7 of 9 surveys. Other covariates showed inconsistent patterns of association with inflammation. In a pooled analysis of surveys that measured malaria, stunting was associated with elevated AGP but not CRP in PSC, and obesity was associated with both elevated CRP and AGP in WRA.

**Conclusions:** Recent morbidity and abnormal anthropometric status are consistently associated with inflammation across a range of environments, whereas more commonly collected demographic covariates were not. Because of the challenge of defining a general demographic population or environmental profile that is more likely to experience inflammation, inflammatory markers should be measured in surveys to account for their effects.

## INTRODUCTION

The acute-phase response (APR) is a sequence of complex physiologic processes that is associated with inflammation and is catalyzed by stress, injury, illness, or trauma. These processes influence a number of mostly short-term biological functions. The main purpose of the inflammatory response is to prevent damage to tissues and to remove harmful molecules and pathogens (1). However, certain chronic conditions such as heart disease, adiposity, and auto-immune diseases as well as fatigue, appetite loss, impaired nutrient absorption, and further dysregulation of steroid-hormonal axes are caused by or consequent to the chronic activation of the inflammatory state (2–4). In many settings, inflammation and malnutrition coexist as part of the 2-way causal malnutrition-infection cycle, whereby undernutrition increases risk and severity of inflammation and infection, and inflammation impairs nutritional status by decreasing food intake and impairing micronutrient absorption (5, 6). The inflammatory process also hinders the assessment of nutritional status by transiently altering nutrient biomarkers (5, 7).

The initiation and coordination of the APR is primarily regulated by the cytokines TNF-α and IL-1 (8). Once the production of cytokines has been initiated, the cytokines, in turn, stimulate the production of acute-phase proteins (APPs), some of which are positive or have a rise in concentration [e.g., C-reactive protein (CRP), α-1-acid glycoprotein (AGP), and ferritin], whereas others are negative or have a decrease in concentration (e.g., retinol-binding protein) (5). These transient shifts in concentration of ferritin and retinol-binding protein, both of which are commonly used biomarkers of micronutrient status, can affect the ability to accurately evaluate an individual’s micronutrient status at the time of concurrent inflammation or micronutrient status at the population level, especially in groups in whom chronic disease or infections are common.

A number of proposed analytic approaches, including the exclusion of individuals with inflammation from final analyses, are designed to adjust micronutrient biomarker concentrations to account for the influence of inflammation on nutritional biomarkers. However, methods such as the exclusion method presume that individuals with inflammation will have the same sociodemographic and health profiles as those of individuals without inflammation; further, these methods require a concurrent APP measurement, which may not always be possible (9, 10). A better understanding of consistent associations between commonly measured covariates and inflammation may facilitate the development of a profile of population segments who are more likely to be experiencing inflammation. For example, a number of studies have shown that adiposity is associated with differential odds of inflammation (11–13). In addition, wasting (which is a measure of acute malnutrition) and stunting (which is a measure of chronic malnutrition) may also be associated with elevated odds of inflammation (14). Socioeconomic status (SES) has also been frequently included as a potential correlate of inflammation with measures of increasing household wealth typically being associated with a reduced likelihood of elevated CRP (15–17).

The objective of this analysis was to investigate the prevalence of inflammation and the strength and overall pattern of associations between inflammation as measured via CRP and AGP and commonly assessed covariates such as household demographics, recent and current illness, and anthropometric status as assessed with the use of cross-sectional data across a range of global environments. Where identified, these associations could be used to inform statistical methods to adjust for inflammation in situations in which biochemical indicators are not measured. Further, these results contribute to the overall Biomarkers Reflecting Inflammation and Nutritional Determinants of Anemia (BRINDA) project to improve our understanding of how to assess iron and vitamin A status during the inflammatory state (18).

## METHODS

### Data sets

We used data from the BRINDA project (www.BRINDA-nutrition.org); the methods for identifying surveys, inclusion and exclusion criteria, and data management have been described in detail elsewhere (18). The BRINDA protocol was reviewed by the Institutional Review Board of the NIH and was deemed non–human subjects research. The surveys included in the BRINDA project were nationally or subnationally representative household-based surveys of apparently healthy individuals and met the following inclusion criteria: *1*) conducted after 2004; *2*) included preschool children (PSC) (age range: 6–59 mo) or nonpregnant women of reproductive age (WRA) (age range: 15–49 y); and *3*) measured ≥1 biomarker of anemia (hemoglobin), iron (ferritin or soluble transferrin receptor) or vitamin A status (retinol-binding protein or retinol) and ≥1 biomarker of inflammation (AGP or CRP). All 16 PSC and 10 WRA BRINDA surveys were used for this analysis. These surveys, which were nationally representative unless otherwise stated, were conducted in Bangladesh (subnational), Cameroon, Cote d’Ivoire, Colombia, Georgia, Kenya (2007 and 2010; both subnational), Laos, Liberia, Mexico (2006 and 2012), Nicaragua, Philippines (subnational), Pakistan, Papua New Guinea (PNG), and the United States. Both CRP and AGP were measured in 9 of 16 PSC surveys. Of the remaining 7 surveys, 5 surveys had CRP data only, whereas 2 surveys had only AGP data. All 10 WRA surveys measured CRP, and 5 surveys further measured AGP. The per capita gross national income (GNI), which was converted to US dollars with the use of the World Bank Atlas method and divided by the midyear population, was obtained for each country reflecting the year that the survey was collected and was used to define low-income (GNI: ≤$1045), lower-middle–income (GNI: $1046–$4125), upper-middle–income (GNI: $4126–$12,735), and high-income (GNI: ≥$12,736) countries (19, 20).

### Laboratory analysis

Venous or capillary blood was collected from each respondent, and serum was stored at −20°C until analysis; the PNG survey used dried blood spots. AGP and CRP concentrations were assessed with the use of a sandwich ELISA at the VitMin Laboratory in 9 of 16 PSC surveys and 4 of 10 WRA surveys (21). In other countries, AGP and CRP were assessed with the use of turbidimetry, nephelometry, and immunoassay methods. Malaria was assessed with the use of microscopy in Kenya and Côte d’Ivoire (22), the Paracheck Pf rapid diagnostic test (Orchid Biomedical System) in Liberia, and plasma histidine-rich protein 2 (Cellabs Pty Ltd.) in Cameroon. The methods for identifying data sets, inclusion and exclusion criteria, and data management for the BRINDA project have been described in the methodologic overview in this supplement, which is an open access publication (23).

### Statistical analyses

Individuals were classified as having inflammation if CRP or AGP concentrations were >5 mg/L or >1 g/L, respectively, which generally reflect acute and longer-term inflammation, respectively (9, 21). Malaria status was dichotomized (positive or negative). Rural or urban residence was defined by the investigators who conducted the survey.

Household SES was defined within each survey on the basis of household income in PNG, the poverty-index ratio in the United States, and an asset score that was based on household ownership of individual items in all other surveys (except in Georgia, where income or assets were not measured). For bivariate analyses, the lowest 2 quintiles were compared with a referent group of all higher quintiles. For PNG, an ordinal income variable with 6 levels was used to create a binary SES variable to compare the lowest 2 levels with the higher 4 levels. For Georgia, only a binary variable was available that represented the number of household members who were employed or earning income. For the United States, a poverty-index ratio of family income to family size was used to create a 5-level variable that was categorized to create a binary variable to compare the 2 lowest levels with the highest 3 levels. SES data were not available from Nicaragua or Bangladesh.

Age was categorized as 6–11, 12–23, 24–35, and 36–59 mo and 15–19, 20–29, 30–39 and 40–49 y. Recent illness (e.g., fever, diarrhea, and cough) was based on a 2-wk recall from similarly worded questionnaires with data availability varying by survey. Caregivers reported recall data for children. Weight and height were measured and used to calculate, for PSC, stunting (height-for-age *z* score <−2 on the basis of WHO 2006 growth standards), wasting (weight-for-height *z* score <−2), and overweight and obesity [children aged <24 mo: weight-for-length *z* score >1 (overweight) or 2 (obese); children aged ≥24 mo: BMI-for-age *z* score >1 (overweight) or >2 (obese)]. For WRA, BMI (in kg/m^2^) was applied to define underweight (<18.5), overweight (25 to <30), and obese (≥30). The breastfeeding status of PSC reflected the caregiver having reported that the child breastfed ≥1 time in the past 24 h. Lactating status for WRA reflected having reported current breastfeeding of any amount.

The majority of analyses were conducted at the survey level. Statistical approaches adjusted for the sampling weight, strata, and cluster (as applicable) to account for the complex survey design as appropriate for each survey analysis. Results from exploratory data analyses described the distribution of data as well as the prevalence of dichotomous variables. A bivariable logistic regression with inflammation assigned as the outcome, with separate models for CRP- and AGP-defined inflammation, was used to determine the unadjusted association between inflammation and demographic, anthropometric, and morbidity variables for covariates that were available in ≥4 surveys. With an objective to achieve consistent models across all surveys, multivariable models were developed to include only those covariates that are commonly measured in surveys: sex (PSC only), age, SES, rural or urban residence, and anthropometric measures [stunting, wasting, high BMI (PSC only), and overweight or obese (WRA only)]. Morbidity information was less consistently collected; the inclusion of morbidity variables (fever, diarrhea, cough, and malaria) in multivariable models did not significantly change the coefficients for any of the adjusted covariates (*P* > 0.05). Consequently, morbidities were not included in the final multivariable models. Another multivariable model was created to include breastfeeding status in addition to the base multivariable model. All multivariable models were assessed for collinearity with the use of condition indexes and variance decomposition proportions; no collinearity was identified. To leverage the availability of multiple data sets, we attempted a pooled analysis; however, heterogeneity was considered too great, and there were inconsistencies in the availability of covariate variables across surveys. Instead, we conducted pooled analyses that were restricted to countries with malaria data to further investigate the role of malaria in inflammation when controlling for sociodemographic and anthropometric measures. All analyses were conducted with the use of SAS 9.4 software (SAS Institute) and accounted for a complex survey design.

## RESULTS

### PSC

Across the 16 surveys, there was a wide distribution of per capita GNI (**Table 1**). Although children of all eligible ages (i.e., 6–59 mo) were enrolled, there was variability in age ranges across the surveys. Bangladesh was unique in enrolling only 6–11-mo-olds. Five countries enrolled children 6–59 mo of age, and 5 countries enrolled only children 12–59 mo of age. Four other countries enrolled children aged 6–24 or –36 mo (Table 1).

**TABLE 1:** Age and prevalence of inflammation in preschool children and nonpregnant women of reproductive age by survey, including gross national incomes: the BRINDA project^1^

				CRP		AGP		CRP or AGP
Survey	GNI, $	*n*	Age,^2^ mean (95% CI)	Median (95% CI)	Elevated, % (95% CI)	Median (95% CI)	Elevated, % (95% CI)	Elevated, % (95% CI)
Preschool children								
Bangladesh	780	1493	7.7 (6, 11)	0.9 (0.0, 18.5)	14.3 (12.5, 16.0)	0.9 (0.6, 1.4)	33.4 (31.0, 35.8)	35.8 (33.4, 38.3)
Cameroon	1160	792	29.6 (12, 59)	2.3 (0.1, 17.7)	37.8 (34.3, 41.3)	0.9 (0.6, 1.4)	39.3 (35.8, 42.9)	48.5 (44.9, 52.1)
Colombia	7140	3866	38.0 (12, 59)	0.2 (0.1, 20.0)	18.8 (17.2, 20.5)	—	—	—
Cote d'Ivoire	980	746	29.8 (6, 59)	2.9 (0.2, 38.1)	40.2 (36.6, 43.9)	1.2 (0.6, 2.1)	64.3 (60.7, 67.9)	67.3 (63.8, 70.9)
Georgia	2820	2142	36.2 (12, 59)	1.0 (0.5, 23.6)	24.7 (22.5, 26.9)	—	—	—
Kenya 2007	730	896	19.3 (6, 36)	1.5 (0.0, 25.8)	27.8 (24.9, 30.7)	1.2 (0.6, 2.2)	64.2 (61.0, 67.3)	66.0 (62.9, 69.1)
Kenya 2010	1000	849	22.2 (6, 35)	2.0 (0.1, 39.7)	34.2 (31.0, 37.3)	1.1 (0.6, 1.8)	60.8 (57.5, 64.1)	62.0 (58.7, 65.2)
Laos	510	481	33.2 (6, 59)	0.6 (0.0, 14.0)	16.6 (12.8, 20.4)	0.9 (0.5, 1.7)	41.7 (36.8, 46.6)	44.0 (39.2, 48.9)
Liberia	320	1434	19.2 (6, 36)	2.0 (0.1, 26.3)	29.5 (26.8, 32.3)	1.0 (0.6, 1.6)	56.2 (53.4, 59.1)	59.1 (56.3, 62.0)
Mexico 2006	8280	1592	43.2 (13, 59)	0.5 (0.1, 9.5)	11.2 (8.9, 13.5)	—	—	—
Mexico 2012	9560	2539	37.0 (12, 59)	0.4 (0.0, 11.0)	11.7 (9.2, 14.1)	—	—	—
Nicaragua	1180	1424	34.2 (6, 59)	—	—	0.8 (0.5, 1.4)	24.0 (20.6, 27.3)	—
Philippines	2640	1767	15.4 (6, 24)	0.7 (0.0, 21.4)	13.9 (11.6, 16.2)	0.8 (0.5, 1.3)	21.2 (18.4, 23.9)	26.0 (23.0, 28.9)
Pakistan	1150	7557	24.4 (6, 59)	—	—	0.9 (0.5, 1.7)	35.5 (34.3, 36.7)	—
Papua New Guinea	700	872	30.6 (6, 59)	2.0 (0.0, 18.3)	31.6 (28.4, 34.8)	1.0 (0.6, 1.6)	54.2 (50.7, 57.6)	57.0 (53.6, 60.5)
United States	46,340	1315	36.3 (12, 59)	0.2 (0.1, 6.8)	6.0 (4.4, 7.7)	—	—	—
Women of reproductive age								
Cameroon	1160	760	26.0 (15, 48)	1.2 (0.1, 12.2)	18.1 (15.3, 20.9)	0.8 (0.5, 1.0)	7.1 (5.2, 9.0)	20.0 (17.1, 23.0)
Cote d'Ivoire	980	834	26.1 (15, 48)	1.6 (0.2, 15.7)	19.6 (16.8, 22.5)	0.8 (0.5, 1.4)	26.7 (23.5, 29.9)	33.6 (30.2, 37.0)
Colombia	7140	9083	27.4 (15, 49)	0.2 (0.1, 17.7)	21.7 (20.6, 22.8)	—	—	—
Georgia	2820	1688	31.7 (15, 49)	1.8 (0.5, 24.1)	29.5 (26.9, 32.0)	—	—	—
Laos	510	816	28.3 (15, 49)	0.4 (0.0, 7.9)	7.9 (5.8, 10.0)	0.7 (0.4, 1.2)	9.3 (7.2, 11.5)	13.9 (11.3, 16.5)
Liberia	320	1942	27.1 (15, 49)	1.0 (0.2, 18.6)	14.2 (12.4, 15.9)	0.7 (0.5, 1.1)	10.2 (8.7, 11.7)	18.4 (16.4, 20.3)
Mexico 2006	8280	3032	31.1 (15, 49)	1.8 (0.2, 11.2)	24.2 (21.6, 26.9)	—	—	—
Mexico 2012	9560	3631	33.5 (15, 49)	1.9 (0.2, 12.8)	20.7 (18.2, 23.2)	—	—	—
Papua New Guinea	700	749	27.7 (15, 49)	0.6 (0.0, 9.0)	10.0 (7.6, 12.3)	0.8 (0.6, 1.3)	21.8 (18.5, 25.1)	24.8 (21.4, 28.3)
United States	46,340	3196	34.5 (15, 49)	1.9 (0.1, 17.1)	25.6 (23.7, 27.6)	—	—	—

1 Elevated CRP was defined as a CRP concentration >5 mg/L. Elevated AGP was defined as an AGP concentration >1 g/L. AGP, α-1-acid glycoprotein; BRINDA, Biomarkers Reflecting Inflammation and Nutritional Determinants of Anemia; CRP, C-reactive protein; GNI, gross national income per capita.

2 Age is given in months for preschool children and in years for women of reproductive age.

The prevalence of elevated CRP concentrations, which was available in 14 surveys, ranged from 6.0% (United States) to 40.2% (Cote d’Ivoire). The prevalence of elevated AGP concentrations, which was available in 11 surveys, was generally higher than that of CRP and varied from 21.2% (Philippines) to 64.2% and 64.3% (Kenya 2007 and Cote d’Ivoire, respectively). When considering elevated CRP or AGP for the 9 surveys that reported both indicators, Cote d’Ivoire had the highest prevalence of any inflammation (67.3%), which was followed closely by Kenya 2007 and 2010; the Philippines had the lowest prevalence of any inflammation (26.0%). Elevated CRP was generally more prevalent in the surveys from low-income and lower-middle–income countries than from upper-middle– and high-income countries [range of elevated CRP: 13.9–37.8% (10 surveys) compared with 6.0–18.8% (4 surveys), respectively]. AGP was not available for upper-middle–income and high-income countries.

Across the 14 surveys in which data regarding rural residences compared with urban residences was available, from 28.1% (Mexico 2012) to 100% (Kenya 2007 and 2010) of participants resided in rural areas (**Table 2**). Mexico 2006 had the lowest prevalence of a 2-wk recall for cough (8.7%), whereas Mexico 2012 had the lowest report of diarrhea (0.9%) with no available data for fever or malaria. In contrast, Liberia had the highest reports of cough and diarrhea at 63.8% and 46.6%, respectively. Liberia also had the highest prevalence of reported 2-wk fever (71.8%). The prevalence of malaria, which was measured in 5 surveys, ranged from 19.7% (Kenya 2007) to 32.5% (Kenya 2010).

**TABLE 2:** Sociodemographic characteristics, reported recent illness, malaria prevalence, anthropometric data, and breastfeeding status in preschool children by survey: the BRINDA project^1^

Survey	Low SES, %	Rural, %	Fever, %	Cough, %	Diarrhea, %	Malaria, %	Overweight or obese, %	Stunted, %	Wasted, %	Breastfeeding, %
Bangladesh	—	—	—	—	—	—	6.0 (4.8, 7.5)	19.5 (16.0, 23.6)	17.8 (15.1, 20.8)	—
Cameroon	43.5 (36.5, 50.8)	41.0 (31.7, 50.9)	—	—	—	25.9 (20.7, 31.9)	22.0 (18.5, 26.0)	31.8 (27.5, 36.3)	3.7 (2.6, 5.3)	16.6 (13.7, 20.0)
Cote d'Ivoire	40.7 (34.3, 47.4)	49.0 (44.4, 53.7)	45.3 (40.1, 50.6)	24.2 (20.0, 29.0)	18.3 (14.8, 22.3)	27.2 (22.6, 32.3)	16.5 (14.0, 19.3)	39.2 (34.6, 44.1)	14.0 (10.0, 19.4)	—
Colombia	51.3 (49.1, 53.4)	29.0 (27.6, 30.4)	—	—	—	—	22.3 (20.6, 24.2)	14.1 (12.6, 15.7)	0.7 (0.4, 1.1)	—
Georgia	18.3 (14.9, 22.2)	52.9 (46.1, 59.5)	13.8 (12.0, 15.9)	22.5 (19.9, 25.4)	6.9 (5.6, 8.6)	—	47.6 (44.3, 50.9)	11.9 (9.3, 15.2)	0.6 (0.3, 1.1)	10.4 (8.7, 12.3)
Kenya 2007	42.4 (37.2, 47.7)	100.0	—	—	—	19.7 (16.1, 23.8)	19.4 (16.7, 22.3)	25.4 (22.7, 28.4)	4.5 (3.0, 6.6)	56.8 (52.4, 61.1)
Kenya 2010	39.9 (34.7, 45.4)	100.0	—	—	—	32.5 (28.5, 36.7)	18.7 (16.2, 21.4)	26.1 (23.1, 29.3)	3.3 (2.1, 5.2)	54.0 (50.0, 57.9)
Laos	61.3 (50.7, 70.9)	86.1 (75.5, 92.6)	18.4 (14.5, 23.0)	26.8 (22.1, 32.0)	17.9 (14.6, 21.7)	—	5.5 (3.4, 8.8)	50.7 (45.2, 56.2)	8.0 (5.3, 12.0)	31.6 (26.4, 37.2)
Liberia	35.9 (29.9, 42.4)	62.5 (59.0, 65.9)	71.8 (68.6, 74.8)	63.8 (60.5, 66.9)	46.6 (43.3, 50.0)	29.4 (26.3, 32.7)	12.7 (10.6, 15.2)	35.1 (32.0, 38.3)	9.2 (7.6, 11.1)	49.5 (46.3, 52.7)
Mexico 2006	65.3 (61.1, 69.3)	47.9 (43.6, 52.2)	—	8.7 (6.9, 10.8)	1.7 (0.9, 3.4)	—	30.0 (26.8, 33.3)	21.7 (18.1, 25.8)	1.3 (0.6, 3.0)	—
Mexico 2012	46.1 (42.4, 49.8)	28.1 (25.4, 31.1)	—	11.0 (9.3, 13.0)	0.9 (0.3, 2.6)	—	28.8 (26.1, 31.7)	15.9 (13.3, 18.9)	0.8 (0.5, 1.5)	14.1 (11.0, 18.1)
Nicaragua	—	43.7 (32.2, 55.9)	—	—	—	—	28.8 (23.0, 35.5)	19.0 (15.2, 23.4)	0.3 (0.1, 0.8)	60.8 (53.0, 68.1)
Philippines	84.4 (81.0, 87.3)	90.9 (89.5, 92.2)	33.4 (30.3, 36.8)	12.3 (10.2, 14.9)	16.8 (14.2, 19.6)	—	10.0 (8.0, 12.6)	26.4 (23.0, 30.2)	5.2 (3.9, 6.9)	44.8 (41.1, 48.5)
Pakistan	43.3 (40.8, 45.8)	70.3 (67.3, 73.1)	—	43.8 (42.1, 45.4)	26.6 (25.1, 28.0)	—	12.5 (11.6, 13.5)	44.3 (42.9, 45.8)	15.4 (14.4, 16.4)	76.1 (74.1, 78.1)
PNG	40.7 (30.1, 52.1)	80.6 (69.8, 88.2)	—	—	—	—	20.7 (17.3, 24.5)	44.2 (39.0, 49.5)	4.3 (2.8, 6.4)	42.6 (38.9, 46.3)
United States	41.0 (35.9, 46.3)	—	—	—	—	—	31.8 (27.5, 36.3)	3.6 (2.2, 5.7)	0.5 (0.2, 1.4)	—

1 All values are means (95% CIs). Fever, cough, and diarrhea represent number of episodes within a 2-wk recall and malaria was determined by parasitemia. Malaria was classified with the use of microscopy in Kenya and Côte d’Ivoire, plasma histidine-rich protein 2 in Cameroon, and a rapid diagnostic test in Liberia. For children ≥24 mo of age, overweight and obese indicated BMI-for-age *z* scores >1 and >2, respectively. For children <24 mo of age, overweight and obese indicated weight-for-length *z* scores >1 and >2, respectively. Breastfeeding was based on a caregiver report that the child breastfed ≥1 time in the past 24 h. BRINDA, Biomarkers Reflecting Inflammation and Nutritional Determinants of Anemia; PNG, Papua New Guinea; SES, socioeconomic status.

The prevalence of abnormal growth varied across surveys. Stunting ranged from 3.6% (United States) to 50.7% (Laos), and wasting ranged from <1% (United States) to 17.8% (Bangladesh) (Table 2). The prevalence of overweight and obesity ranged from 5.5% (Laos) to 47.6% (Georgia). Current breastfeeding ranged from 10.4% (Georgia) to 76.1% (Pakistan).

Unadjusted bivariable analyses for recent illness and malaria are shown in **Table 3**. There was a consistent and significant association between elevated CRP and recent fever and malaria in all 5 surveys with relevant data. In contrast, recent cough and diarrhea were positively associated with elevated AGP in most surveys along with fever and malaria in all surveys. Adjustment for demographics and anthropometric measures did not substantially change these associations (data not shown).

**TABLE 3:** Unadjusted ORs of elevated acute-phase protein concentrations given recent illness or malaria in preschool children and nonpregnant women of reproductive age by survey: the BRINDA project^1^

Survey	Fever	Cough	Diarrhea	Malaria
CRP concentration >5 mg/L				
Preschool children				
Cameroon	—	—	—	7.43 (5.2, 10.57)**
Cote d'Ivoire	1.67 (1.25, 2.22)**	1.48 (1.05, 2.10)*	1.06 (0.73, 1.53)	4.96 (3.20, 7.68)**
Colombia	—	—	—	—
Georgia	2.43 (1.83, 3.24)**	1.72 (1.31, 2.27)**	1.34 (0.85, 2.12)	—
Kenya 2007	—	—	—	3.99 (2.86, 5.57)**
Kenya 2010	—	—	—	7.53 (5.09, 11.13)**
Laos	2.43 (1.50, 3.94)**	1.46 (0.82, 2.61)	1.55 (0.92, 2.62)	—
Liberia	2.15 (1.58, 2.92)**	1.16 (0.89, 1.51)	1.04 (0.79, 1.36)	3.43 (2.65, 4.43)**
Mexico 2006	—	1.33 (0.67, 2.64)	0.39 (0.05, 3.23)	—
Mexico 2012	—	1.30 (0.75, 2.25)	11.52 (1.68, 79.15)*	—
Nicaragua	—	—	—	—
Philippines	2.21 (1.57, 3.13)**	1.11 (0.70, 1.77)	1.02 (0.58, 1.82)	—
Women of reproductive age				
Cameroon	—	—	—	2.88 (1.94, 4.28)**
Cote d'Ivoire	1.36 (0.84, 2.20)	1.48 (0.89, 2.45)	2.11 (1.26, 3.55)**	3.88 (2.04, 7.41)**
Liberia	—	—	—	3.42 (2.52, 4.64)**
Mexico 2006	—	—	2.14 (0.21, 21.52)	—
Mexico 2012	—	1.04 (0.56, 1.94)	2.00 (0.34, 11.63)	—
AGP concentration >1 g/L				
Preschool children				
Cameroon	—	—	—	7.33 (4.93, 10.88)**
Cote d'Ivoire	2.07 (1.56, 2.75)**	1.38 (1.06, 1.80)*	1.72 (1.22, 2.44)**	3.72 (2.44, 5.69)**
Kenya 2007	—	—	—	4.41 (2.63, 7.40)**
Kenya 2010	—	—	—	7.40 (4.94, 11.09)**
Laos	3.87 (2.23, 6.73)**	1.72 (1.15, 2.58)*	2.18 (1.38, 3.44)**	—
Liberia	1.34 (1.04, 1.73)*	1.15 (0.89, 1.48)	1.46 (1.17, 1.80)**	3.12 (2.34, 4.17)**
Philippines	3.01 (2.07, 4.39)**	1.15 (0.74, 1.77)	1.36 (0.93, 2.00)	—
Pakistan	—	1.28 (1.13, 1.44)**	1.27 (1.13, 1.43)**	—
Women of reproductive age				
Cameroon	—	—	—	3.25 (1.85, 5.70)**
Cote d'Ivoire	1.91 (1.32, 2.77)**	1.86 (1.14, 3.01)*	1.91 (1.09, 3.35)*	2.98 (1.52, 5.84)**
Liberia	—	—	—	2.19 (1.49, 3.22)**

1 All values are ORs (Wald’s 95% CIs). Values were computed within each survey separately while accounting for a complex survey design (clustering, weighting, and stratification). Fever, cough, and diarrhea represent number of episodes within a 2-wk recall, and malaria was determined by parasitemia. Malaria was classified with the use of microscopy in Kenya and Côte d’Ivoire, plasma histidine-rich protein 2 in Cameroon, and a rapid diagnostic test in Liberia. Surveys that are not listed did not have data available. *P < 0.05, **P < 0.01. AGP, α-1-acid glycoprotein, BRINDA, Biomarkers Reflecting Inflammation and Nutritional Determinants of Anemia; CRP, C-reactive protein.

Results from multivariable analyses are presented in **Table 4**. Sex was not associated with inflammation except in Laos, where males had a significantly lower prevalence of elevated AGP (*P* < 0.05). With few exceptions, age in months, as a continuous variable, was not linearly associated with inflammation. However, when categorically defined, older age (36–59 mo) compared with 12–23 mo of age was consistently negatively associated with elevated AGP in all 6 surveys with available data reflecting an overall 30–57% decrease in odds of elevated AGP (data not shown). Colombia and Laos showed decreased odds of elevated CRP for the 36–59-mo age group than for 12–23-mo-olds [ORs (95% CIs): 0.57 (0.43, 0.75) and 0.48 (0.24, 0.94), respectively], whereas Georgia and Mexico 2012 showed increased odds of elevated CRP [ORs (95% CIs): 1.33 (1.01, 1.74) and 1.68 (1.00, 2.82), respectively].

**TABLE 4:** Adjusted ORs of elevated acute-phase protein concentrations given demographic characteristics and anthropometric measures in preschool children by survey: the BRINDA project^1^

Survey	Sex	Age, mo	Rural	SES	Stunting	Wasting	Overweight
CRP concentration >5 mg/L							
Bangladesh	0.86 (0.57, 1.28)	0.95 (0.84, 1.08)	—	—	1.00 (0.69, 1.44)	1.24 (0.88, 1.74)	0.86 (0.44, 1.68)
Cameroon	1.22 (0.89, 1.68)	0.99 (0.97, 1.00)	0.77 (0.46, 1.31)	2.05 (1.27, 3.31)**	1.16 (0.75, 1.79)	0.43* (0.19, 0.99)*	0.98 (0.67, 1.43)
Cote d'Ivoire	0.77 (0.55, 1.06)	1.00 (0.99, 1.01)	1.76 (1.21, 2.57)**	1.49 (1.07, 2.08)*	1.13 (0.79, 1.63)	1.21 (0.81, 1.82)	0.95 (0.55, 1.63)
Colombia	0.87 (0.70, 1.08)	0.99 (0.98, 0.99)	0.77 (0.58, 1.03)	1.14 (0.87, 1.5)	1.23 (0.91, 1.67)	0.81 (0.28, 2.32)	1.06 (0.82, 1.38)
Georgia	0.86 (0.68, 1.09)	1.01 (1.00, 1.02)	0.90 (0.67, 1.21)	1.03 (0.78, 1.38)	1.37 (0.88, 2.11)	1.93 (0.54, 6.83)	0.94 (0.74, 1.21)
Kenya 2007	0.90 (0.71, 1.15)	1.01 (0.99, 1.03)	—	1.02 (0.73, 1.43)	0.86 (0.56, 1.30)	2.06 (0.94, 4.51)	0.93 (0.6, 1.47)
Kenya 2010	0.78 (0.57, 1.05)	1.00 (0.98, 1.02)	—	1.07 (0.79, 1.45)	1.10 (0.79, 1.53)	3.04 (1.06, 8.69)	1.24 (0.87, 1.77)
Laos	0.90 (0.49, 1.67)	0.97 (0.95, 0.99)	0.48 (0.19, 1.18)	1.26 (0.69, 2.27)	1.50 (0.64, 3.54)	2.01 (0.87, 4.62)	0.48 (0.09, 2.55)
Liberia	1.10 (0.83, 1.47)	1.02 (1.00, 1.03)	2.04 (1.44, 2.90)**	1.12 (0.79, 1.57)	1.21 (0.93, 1.57)	1.42 (0.89, 2.28)	1.13 (0.73, 1.75)
Mexico 2006	1.05 (0.67, 1.64)	0.99 (0.97, 1.00)	1.01 (0.64, 1.59)	1.22 (0.72, 2.07)	1.45 (0.82, 2.57)	1.42 (0.30, 6.73)	1.21 (0.72, 2.05)
Mexico 2012	1.37 (0.86, 2.20)	1.01 (0.99, 1.03)	0.70 (0.47, 1.05)	0.57 (0.35, 0.93)*	2.34 (1.23, 4.46)**	0.70 (0.09, 5.58)	0.65 (0.39, 1.09)
Philippines	1.05 (0.84, 1.48)	0.97 (0.94, 1.01)	0.65 (0.46, 0.94)*	1.68 (1.00, 2.82)	0.74 (0.45, 1.21)	1.29 (0.56, 2.97)	0.71 (0.37, 1.38)
Papua New Guinea	1.13 (0.83, 1.53)	0.99 (0.98, 1.00)**	1.05 (0.52, 2.09)	1.00 (0.63, 1.61)	2.26 (1.62, 3.16)**	1.35 (0.71, 2.56)	0.53 (0.32, 0.86)**
United States	0.66 (0.36, 1.20)	0.99 (0.96, 1.01)	—	1.16 (0.69, 1.98)	0.20 (0.03, 1.46)	—	1.01 (0.54, 1.88)
AGP concentration >1 g/L							
Bangladesh	0.96 (0.72, 1.28)	1.01 (0.93, 1.10)	—	—	1.39 (1.13, 1.71)**	1.39 (1.06, 1.82)*	1.16 (0.7, 1.92)
Cameroon	1.02 (0.74, 1.40)	0.99 (0.97, 1.00)	0.60 (0.33, 1.09)	2.15 (1.25, 3.71)**	2.05 (1.41, 2.99)**	0.52 (0.21, 1.28)	0.84 (0.56, 1.25)
Cote d'Ivoire	0.85 (0.61, 1.17)	0.99 (0.98, 1.00)	2.09 (1.38, 3.17)**	1.28 (0.81, 2.02)	1.46 (1.05, 2.02)*	1.58 (1.00, 2.50)	1.03 (0.58, 1.81)
Kenya 2007	1.01 (0.75, 1.37)	1.00 (0.98, 1.02)	—	1.12 (0.85, 1.49)	1.71 (1.21, 2.41)**	1.81 (0.77, 4.25)	1.25 (0.85, 1.83)
Kenya 2010	0.83 (0.63, 1.09)	1.00 (0.99, 1.02)	—	1.37 (1.02, 1.85)*	1.45 (1.05, 2.00)*	5.60 (1.69, 18.57)**	1.25 (0.91, 1.71)
Laos	1.56 (1.07, 2.26)*	0.98 (0.97, 0.99)	1.04 (0.51, 2.12)	1.06 (0.62, 1.79)	1.02 (0.65, 1.59)	1.51 (0.65, 3.49)	0.53 (0.18, 1.53)
Liberia	0.87 (0.68, 1.11)	1.02 (1.00, 1.03)*	1.55 (1.14, 2.11)**	1.3 (0.94, 1.79)	1.62 (1.23, 2.13)**	1.81 (1.23, 2.66)**	0.95 (0.64, 1.42)
Philippines	1.04 (0.75, 1.44)	1.01 (0.98, 1.04)	0.69 (0.50, 0.97)**	1.43 (0.91, 2.23)	1.28 (0.80, 2.06)	1.14 (0.55, 2.37)	0.69 (0.40, 1.19)
Pakistan	0.89 (0.80, 1.00)	0.99 (0.99, 0.99)	1.25 (1.09, 1.44)**	1.24 (1.09, 1.42)**	1.12 (1.00, 1.26)	0.98 (0.84, 1.13)	1.07 (0.90, 1.29)
Papua New Guinea	0.96 (0.72, 1.29)	0.98 (0.98, 0.99)**	0.90 (0.52, 1.56)	0.86 (0.56, 1.32)	1.65 (1.20, 2.27)**	2.09 (0.87, 5.05)	0.85 (0.56, 1.28)

1 All values are ORs (95% CIs). Values were adjusted for all variables in the row. Data of rural residence compared with urban residence were based on a report by survey implementers. Household SES was classified as low, medium, or high on the basis of household member employment in Georgia, household income in Papua New Guinea, the poverty-index ratio in the United States, and an asset score in all other surveys; for bivariate analyses, the low-SES group was compared with a reference group of medium-SES and high-SES combined. For children ≥24 mo of age, overweight indicated a BMI-for-age *z* score >1 compared with normal BMI for age as determined with the use of a WHO macro. For children <24 mo of age, overweight indicated a weight-for-length *z* score >1 compared with normal weight for length (WHO 2006). Breastfeeding was based on a caregiver report that the child breastfed ≥1 time in the past 24 h. *P < 0.05, **P < 0.01. AGP, α-1-acid glycoprotein; BRINDA, Biomarkers Reflecting Inflammation and Nutritional Determinants of Anemia; CRP, C-reactive protein; SES, socioeconomic status.

Of the significant results, rural residence was most commonly associated with increased odds of elevated APPs (Table 4). Specifically, rural residence was positively associated with elevated APPs in Pakistan (only AGP available) and Cote d’Ivoire and Liberia (both elevated CRP and AGP were available). In contrast, urban residence was associated with both elevated APPs in the Philippines. Low SES was positively associated with increased odds of elevated CRP in 2 of 13 surveys and with elevated AGP in 3 of 9 surveys.

Although stunting was significantly associated with elevated CRP in only 2 (Mexico 2012 and PNG) of 14 surveys, stunting was significantly, positively associated with elevated AGP in 7 of 10 surveys (Table 4). Both surveys with a significant association between stunting and elevated CRP also showed a significant association with elevated AGP. Wasting was less commonly associated with elevated APPs such that none of the surveys showed an association with elevated CRP, and 3 of 10 surveys showed a significant, positive association with elevated AGP.

Current breastfeeding status was not associated with elevated CRP in any survey (**Supplemental Table 1**). However, current breastfeeding was associated with a decreased prevalence of elevated AGP in 2 (Kenya 2007 and Liberia) of 9 surveys and with an increased prevalence of elevated AGP in 1 survey (Philippines). Associations between other covariates and inflammation did not change substantially after adjusting for breastfeeding.

Pooled analyses of countries that measured malaria infection (Cameroon, Cote d’Ivoire, Kenya 2007, Kenya 2010, and Liberia) showed similar results as those of survey-stratified analyses. Malaria and wasting were strongly associated with both elevated CRP and elevated AGP (**Supplemental Table 2**). Stunting was positively associated with AGP but not with CRP. Older age and breastfeeding were protective for elevated AGP but not for elevated CRP.

### WRA

The mean age of participating women ranged from ∼26 to 35 y (Table 1). The prevalence of elevated CRP was lowest in Laos (7.9%) and highest in Georgia (29.5%). Cameroon reported the lowest prevalence of elevated AGP (7.1%), whereas Cote d’Ivoire had the highest prevalence of elevated AGP (26.7%). For the 4 surveys that reported both CRP and AGP, Laos reported the lowest prevalence of any inflammation (13.9%), and Cote d’Ivoire had the highest prevalence of any inflammation (33.6%). Elevated CRP was generally more prevalent in the surveys from middle-, high-middle–, and high-income countries than from low-income countries [range of elevated CRP: 18.1–29.5% (6 surveys) compared with 7.9–19.6% (4 surveys), respectively]. AGP was not available for upper-middle– and high-income countries.

Most women in Liberia (70.2%) and Laos (67.9%) resided in rural households, whereas only ∼20% of women in Colombia and Mexico lived in rural areas (**Table 5**). Malaria was less frequent in women than in children and ranged from 5% of women in Cote d’Ivoire to nearly 30% of women in PNG. Surveys that reported the highest prevalence of obesity included Mexico 2012 (36.3%), the United States (30.8%), and Mexico 2006 (28.7%), whereas surveys that reported the lowest prevalence of obesity included Laos, PNG, and Cote d’Ivoire (3.1%, 5.4%, and 7.2%, respectively).

**TABLE 5:** Sociodemographic characteristics, malaria prevalence, anthropometric data, and lactation status in women of reproductive age by survey: the BRINDA project^1^

Survey	Low SES	Rural	Malaria	Underweight	Overweight	Obese	Lactating
Cameroon	43.0 (39.2, 46.7)	41.3 (37.6, 44.9)	15.0 (12.3, 17.7)	8.4 (6.4, 10.4)	22.1 (19.0, 25.2)	10.7 (8.5, 13.0)	18.0 (14.8, 21.2)
Cote d'Ivoire	38.6 (35.2, 42.1)	46.6 (43.0, 50.1)	5.0 (3.3, 6.6)	9.0 (7.0, 11.0)	16.3 (13.6, 18.9)	7.2 (5.4, 9.1)	—
Colombia	37.1 (35.9, 38.4)	21.5 (20.4, 22.6)	—	6.3 (5.6, 6.9)	28.1 (26.8, 29.3)	13.3 (12.4, 14.2)	8.9 (8.1, 9.7)
Georgia	19.1 (15.8, 22.9)	50.2 (47.5, 53.0)	—	5.0 (3.8, 6.3)	24.9 (22.6, 27.2)	19.1 (16.9, 21.3)	8.4 (6.7, 10.1)
Laos	38.6 (34.9, 42.2)	67.9 (64.1, 71.7)	—	14.5 (11.8, 17.2)	11.5 (9.0, 14.1)	3.1 (1.6, 4.6)	—
Liberia	33.9 (31.5, 36.4)	70.2 (68.3, 72.1)	17.9 (15.8, 19.9)	—	—	—	—
Mexico 2006	46.6 (43.5, 49.6)	28.7 (26.0, 31.4)	—	2.9 (1.8, 4.0)	34.7 (31.6, 37.8)	28.7 (25.9, 31.4)	—
Mexico 2012	30.4 (27.6, 33.1)	21.8 (19.6, 24.1)	—	1.4 (0.8, 2.1)	34.7 (31.5, 37.9)	36.3 (33.1, 39.4)	—
Papua New Guinea	39.2 (28,3, 51.2)	78.5 (75.4, 81.6)	29.9 (22.6, 37.1)	5.2 (3.6, 6.7)	17.8 (14.6, 20.9)	5.4 (3.7, 7.1)	—
United States	26.2 (24.4, 28.0)	—	—	3.4 (2.6, 4.2)	23.6 (21.7, 25.6)	30.8 (28.7, 32.9)	29.9 (22.6, 37.1)

1 All values are means (95% CIs). Malaria was determined by parasitemia. Malaria was classified with the use of microscopy in Côte d’Ivoire, plasma histidine-rich protein 2 in Cameroon, and a rapid diagnostic test in Liberia. Underweight, overweight, and obese were defined as BMI (in kg/m^2^) <18.5, 25 to <30, and ≥30, respectively. Lactating was based on a respondent report of breastfeeding currently or within the past 24 h. BRINDA, Biomarkers Reflecting Inflammation and Nutritional Determinants of Anemia; SES, socioeconomic status.

Unadjusted bivariable analyses between inflammation and recent illness and malaria are presented in Table 3. There was a consistent and significant association between elevated CRP and malaria with all 3 surveys reporting data that revealed significant results (with ORs ranging from 2.9 to 3.9, *P* < 0.05). Of recent illnesses, only diarrhea was significantly associated with elevated CRP (in Cote d’Ivoire). Only Cote d’Ivoire had both AGP and recent-illness data available, but all observed associations were significant. Malaria was also significantly associated with elevated AGP in all 3 countries with available data.

Results from multivariable analyses are presented in **Table 6**. Age was significantly positively associated with elevated CRP in 4 countries with available data and significantly inversely associated with elevated CRP in 1 survey. In contrast, age was not significantly associated with elevated AGP with the exception of PNG where there was a small inverse association. Rural location was significantly inversely associated with elevated CRP in only one survey with available data (Colombia) but was not associated with elevated AGP. SES was not significantly associated with elevated CRP or AGP.

**TABLE 6:** Adjusted ORs of elevated acute-phase protein concentrations given demographic characteristics and anthropometry in nonpregnant women of reproductive age by survey: the BRINDA project^1^

Survey	Age	Rural	SES	Underweight	Overweight	Obese
CRP concentration >5 mg/L						
Cameroon	0.99 (0.97, 1.03)	1.19 (0.76, 1.86)	0.88 (0.59, 1.31)	1.08 (0.53, 2.19)	0.56 (0.34, 0.93)*	1.37 (0.79, 2.37)
Cote d'Ivoire	0.97 (0.95, 1.00)*	0.85 (0.48, 1.50)	0.96 (0.54, 1.7)	1.03 (0.52, 2.03)	1.45 (0.87, 2.43)	3.36 (1.83, 6.17)**
Colombia	1.01 (1.00, 1.02)*	0.78 (0.63, 0.97)*	1.13 (0.94, 1.35)	0.97 (0.71, 1.33)	1.60 (1.35, 1.90)**	3.26 (2.67, 3.99)**
Georgia	1.01 (1.00, 1.02)	1.13 (0.82, 1.56)	0.86 (0.61, 1.21)	0.23 (0.09, 0.59)**	1.85 (1.37, 2.51)**	4.50 (3.03, 6.67)**
Laos	1.04 (1.01, 1.10)**	0.90 (0.42, 1.97)	1.01 (0.45, 2.22)	1.72 (0.74, 3.99)	4.11 (1.83, 9.20)**	2.59 (0.82, 8.11)
Liberia	1.01 (0.99, 1.02)	0.84 (0.58, 1.23)	0.98 (0.63, 1.53)	—	—	—
Mexico 2006	1.04 (1.02, 1.05)**	1.06 (0.78, 1.46)	0.89 (0.66, 1.21)	0.97 (0.33, 2.87)	1.80 (1.14, 2.84)**	5.71 (3.71, 8.80)**
Mexico 2012	1.00 (0.97, 1.03)	0.75 (0.55, 1.03)	1.03 (0.75, 1.42)	0.04 (0.01, 0.33)**	1.98 (1.21, 3.23)**	6.04 (3.85, 9.47)**
Papua New Guinea	1.00 (0.97, 1.02)	1.04 (0.49, 2.22)	1.03 (0.53, 1.98)	2.20 (0.99, 4.88)	1.06 (0.51, 2.17)	3.28 (1.35, 7.96)**
United States	1.02 (1.01, 1.03)**	—	1.21 (0.96, 1.52)	0.15 (0.06, 0.38)**	2.57 (1.77, 3.73)**	8.46 (6.31, 11.34)**
AGP concentration >1 g/L						
Cameroon	1.00 (0.96, 1.04)	1.92 (0.93, 3.93)	0.78 (0.41, 1.50)	1.31 (0.44, 3.93)	0.48 (0.18, 1.30)	1.09 (0.43, 2.75)
Cote d'Ivoire	0.99 (0.97, 1.01)	1.35 (0.83, 2.19)	0.87 (0.57, 1.33)	1.50 (0.77, 2.93)	0.71 (0.41, 1.22)	5.03 (2.76, 9.17)**
Laos	0.98 (0.96, 1.01)	1.91 (0.94, 3.87)	1.17 (0.61, 2.24)	1.29 (0.65, 2.59)	2.48 (1.03, 6.0.00)*	3.14 (0.87, 11.3)
Liberia	0.99 (0.97, 1.01)	1.15 (0.78, 1.68)	0.75 (0.49, 1.17)	—	—	NA
Papua New Guinea	0.97 (0.95, 0.99)*	0.77 (0.45, 1.35)	1.60 (1.00, 2.55)	1.72 (0.69, 4.26)	0.87 (0.55, 1.38)	2.58 (1.15, 5.79)*

1 All values are ORs (95% CIs). Values were adjusted for all variables in the row. Data of rural residence compared with urban were based on a report by survey implementers. Household SES was classified as low, medium, or high on the basis of household member employment in Georgia, household income in Papua New Guinea, the poverty-index ratio in the United States, and an asset score in all other surveys; for bivariate analyses, the low-SES group was compared with a reference group of medium-SES and high-SES combined. Underweight, overweight, and obese were defined as BMI (in kg/m^2^) <18.5, 25 to <30, and ≥30, respectively. Lactating was based on a respondent report of breastfeeding currently or within the past 24 h. *P < 0.05, **P < 0.01. AGP, α-1-acid glycoprotein; BRINDA, Biomarkers Reflecting Inflammation and Nutritional Determinants of Anemia; CRP, C-reactive protein; NA, not applicable; SES, socioeconomic status.

Compared with normal BMI (18.5–24), low BMI was associated with lower odds of elevated CRP in the 3 surveys with significant results (Table 6). Data from women in 6 of 9 surveys showed a positive association between overweight (25 to <30) relative to normal BMI, and only 1 survey (Cameroon) showed the opposite association. However, when obese participants were compared with participants with normal BMI, data from women in 7 of 9 countries showed a significant, positive association with elevated CRP with ORs (95% CIs) ranging from 3.26 (2.67, 3.99) (Colombia) to 8.46 (6.31, 11.34) (United States). In contrast, BMI was only associated with AGP-defined inflammation in 3 surveys. Current lactation was significantly associated with elevated CRP in only 1 (Colombia) of 3 surveys with available data (**Supplemental Table 3**). There was no association between lactation and elevated AGP.

Pooled analyses of surveys in WRA that measured malaria infection (Cameroon, Cote d’Ivoire, and Liberia) included only malaria, age, rural location compared with urban location, and SES, because other variables were not consistently available in all surveys. Malaria was significantly associated with elevated CRP and elevated AGP, whereas rural location was significantly associated with elevated AGP but not with elevated CRP (**Supplemental Table 4**). In a pooled analysis of the 2 malarial countries that measured anthropometric measures (Cameroon, Cote d’Ivoire), obesity and malaria were significantly associated with both elevated CRP and AGP (data not shown).

## DISCUSSION

To our knowledge, our study is the largest multicountry analysis to evaluate the prevalence of acute and long-term inflammation and factors associated with inflammation according to survey and population group. Data from ∼30,000 PSC and 25,000 WRA showed an overall high prevalence of inflammation with a median of any inflammation of 57.0% in PSC and of 20.0% in WRA. Although there was considerable heterogeneity in the prevalence of inflammation, inflammation was generally more common in low-income and lower-middle–income countries (11 of 14 countries) in PSC and was more common in higher-income countries in WRA (20). AGP is characterized as a measure of long-term inflammation, and the association between AGP and stunting and more common illnesses including diarrhea in the low- and lower-middle–income countries is suggestive of recurrent exposure to environmental factors promoting an APR (24).

In PSC, we showed a more-consistent and higher magnitude of significant positive associations between stunting and elevated AGP than between stunting and elevated CRP, which potentially reflects AGP’s role as a marker of long-term inflammation as opposed to acute inflammation (7, 24, 25). More specifically, stunting is usually associated with long-term exposure to a poor diet or repeated infection episodes that limit critical catch-up growth until 15–18 mo of age (26, 27). In PSC, elevated CRP was only associated with fever and malaria, whereas elevated AGP was associated with fever, cough, diarrhea, and malaria in a majority of data sets. In the pooled analysis in surveys that measured malaria infection, stunting was significantly positively associated only with elevated AGP, whereas wasting was significantly associated with elevated concentrations of both CRP and AGP.

In WRA, the factor that was most consistently associated with elevated CRP was obesity (significantly positively associated in 7 of 9 countries with data) as has been shown in many previous studies (12). Obesity was also significantly associated with elevated AGP concentrations in 2 of 4 countries with available data. These trends were also seen with overweight. Pooled analyses of surveys that assessed malaria infection also showed a significant relation of obesity with both biomarkers of inflammation although only 2 countries contributed data. This analysis contributes to the mounting body of literature describing the association between obesity and inflammation by showing consistent patterns across a variety of countries and environments.

With regard to recent illness, limited data were available in WRA. The analyses of the PSC data showed a consistent, strong, positive association between recent fever and malaria and any inflammation. The association of malaria and inflammation was confirmed in pooled analyses; unfortunately, fever could not be included because of inconsistencies in the data collection. In contrast, the association between a recent cough and diarrhea and inflammation differed by APPs. These 2 morbidities, cough and diarrhea, were generally not significantly associated with elevated CRP (data available in 7 surveys) but were significantly, positively associated with elevated AGP in a majority of the 5 surveys with data. These findings suggest that reports of recent cough and diarrhea may have reflected a state of long-term infection or that the children were going through the convalescent stage of recovery (normal CRP with elevated AGP) (9).

Country-level analyses, which included age as a continuous variable, did not show consistent associations across the age distribution with elevated CRP or AGP. The pooled analysis, which was conducted in surveys with malaria data, showed a weak but significant negative relation of AGP with continuous age. In other analyses that incorporated age as a categorical variable, older age (36–59 mo), compared with children aged 12–23 mo, was associated with a 30–57% decrease in odds of elevated AGP before adjustment for other covariates (data not shown). Similarly, in a recent cohort study of infants in Bolivia, CRP and AGP concentrations increased with age from 2 to 18 mo, and in a national, cross-sectional survey in Tanzania, children aged 36–59 mo were less likely to have elevated CRP than were children aged 6–35 mo (15, 28). Younger children have an immature adaptive immune system and receive maternal antibodies through breastmilk for the first 6–12 mo of age (29). In addition, exposure to environmental pathogens can increase, fairly drastically, as toddlers gain mobility and begin to eat more complementary foods including foods prepared with unclean water or that are stored in unhygienic conditions (30). When coupled with a more mature immune system, older PSC (aged 36–59 mo) are more able to combat the continuous exposure to environmental pathogens that are reflected by the pattern of association with elevated AGP. These patterns are consistent with results suggesting a peak in inflammation between 12 and 35 mo of age.

We showed limited consistent patterns in the association between commonly measured sociodemographic factors and elevated CRP and AGP. Within surveys, there was a similar pattern of an association between SES and both measures of inflammation, but the pattern varied inconsistently between surveys probably because of the different environmental factors within each environment (e.g., malaria endemicity, variable measures for SES, and the GNI of the country, which may reflect the availability of health care and other public health interventions). Rural residence compared with urban residence showed a mixed pattern of an association with inflammation as well. Cote d’Ivoire, Liberia, and Pakistan are all countries where malaria is present, and rural communities tend to have higher parasitemia than in urban communities, which is probably reflected in the association between rural living and elevated CRP and AGP concentrations (31). The Philippines and Mexico do not have endemic malaria; the relation between the APPs and rural residence compared with urban residence could reflect differential SES distributions. Although there are not sufficient data available to explore these associations and possible confounders in more depth, these patterns reveal a challenge with defining a consistent population or environment profile in which women and children may have higher odds of inflammation.

The strengths of the analysis include the use of data from a number of WHO regions and the incorporation of the concurrent measurements of AGP, which is an uncommonly measured inflammatory biomarker that reflects long-term inflammation, CRP, which is a more commonly measured biomarker that predominantly reflects acute inflammation, and a number of covariates. The data analysis is limited by inconsistent data-collection methods across the multiple, cross-sectional surveys, which is reflected by different age (PSC only), rural compared with urban, and SES distributions across surveys, and recent illness being unavailable for 8 surveys in PSC and 7 surveys in WRA. In addition, a number of important covariates of inflammation that were not consistently measured in the surveys included indicators of access to clean water and sanitation and nonmalarial infections (e.g., HIV and soil-transmitted helminths). We did not include pregnant women in our analysis, which is a research gap because of the shown increase in inflammatory proteins in pregnancy and their potential link to adverse maternal and infant health outcomes (32, 33). Further, breastfeeding and lactation were included as binary variables across the full age distribution and did not account for a differential association across the age distribution. In addition, the indicator did not account for the history of breastfeeding. Finally, these results are limited to associations, rather than causations, between covariates and inflammation. We limited the scope of this paper to evaluate the prevalence of inflammation in different settings and to see if factors that are associated with elevated CRP and AGP are consistent across environments and population characteristics. We also used standard cutoffs for CRP and AGP to define inflammation; analyses with the use of lower cutoffs or a continuous distribution of CRP and AGP may produce different results. However, a recent study in Bolivian infants examined factors that are associated with inflammation with the use of CRP concentration cutoffs of 5, 3, and 1.1 mg/L, and the results were similar (28).

In conclusion, strong and consistent patterns of association between elevated CRP or AGP and covariates were limited to positive associations with recent fever and malaria (CRP and AGP), recent diarrhea, stunting, and elevated AGP in PSC and with overweight and obesity and elevated CRP in WRA. These findings support the well-known association between recent and current morbidity and overweight and obesity and the inflammatory response while providing support for an association between stunting and longer-term inflammation across a range of environments and populations. However, there are no consistent associations with demographic covariates across the surveys. Taken together, these results, as summarized across a variety of environments, suggest that no clear profile of demographic characteristics such as sex, SES, or rural residence are likely to be associated with elevated inflammation while, at the same time, providing evidence that the exclusion method would disproportionately exclude individuals experiencing certain morbidities or abnormal growth. Because of these relations between commonly measured covariates and inflammation, as defined by elevated AGP or CRP, the concurrent measurement of biochemical inflammatory biomarkers is needed to facilitate the adjustment of nutrition biomarkers that are influenced by inflammation.

## References

[1] Northrop-Clewes CA Interpreting indicators of iron status during an acute phase response–lessons from malaria and human immunodeficiency virus. Ann Clin Biochem 2008;45:18–32. 18275670 10.1258/acb.2007.007167

[2] Rytter MJ, Kolte L, Briend A, Friis H, Christensen VB The immune system in children with malnutrition–a systematic review. PLoS One 2014;9:e105017. 25153531 10.1371/journal.pone.0105017 PMC4143239

[3] Straub RH, Schradin C Chronic inflammatory systemic diseases: an evolutionary trade-off between acutely beneficial but chronically harmful programs. Evol Med Public Health 2016;2016:37–51. 26817483 10.1093/emph/eow001 PMC4753361

[4] Rubio-Ruiz ME, Peredo-Escarcega AE, Cano-Martinez A, Guarner-Lans V An evolutionary perspective of nutrition and inflammation as mechanisms of cardiovascular disease. Int J Evol Biol 2015;2015:179791. 26693381 10.1155/2015/179791 PMC4677015

[5] Raiten DJ, Sakr Ashour FA, Ross AC, Meydani SN, Dawson HD, Stephensen CB, Brabin BJ, Suchdev PS, van Ommen B, INSPIRE Consultative Group. Inflammation and Nutritional Science for Programs/Policies and Interpretation of Research Evidence (INSPIRE). J Nutr 2015;145:1039S–108S. 25833893 10.3945/jn.114.194571 PMC4448820

[6] Suchdev PS, Boivin MJ, Forsyth BW, Georgieff MK, Guerrant RL, Nelson CA III Assessment of nuerodevelopment, nutrition, and inflammation from fetal life to adolescence in low-resource settings. Pediatrics 2017;139(Suppl 1). 10.1542/peds.2016-2828E 28562246

[7] Bresnahan KA, Tanumihardjo SA Undernutrition, the acute phase response to infection, and its effects on micronutrient status indicators. Adv Nutr 2014;5:702–11. 25398733 10.3945/an.114.006361 PMC4224207

[8] Feelders RA, Vreugdenhil G, Eggermont AM, Kuiper-Kramer PA, van Eijk HG, Swaak AJ Regulation of iron metabolism in the acute-phase response: interferon gamma and tumour necrosis factor alpha induce hypoferraemia, ferritin production and a decrease in circulating transferrin receptors in cancer patients. Eur J Clin Invest 1998;28:520–7. 9726030 10.1046/j.1365-2362.1998.00323.x

[9] Thurnham DI, McCabe LD, Haldar S, Wieringa FT, Northrop-Clewes CA, McCabe GP Adjusting plasma ferritin concentrations to remove the effects of subclinical inflammation in the assessment of iron deficiency: a meta-analysis. Am J Clin Nutr 2010;92:546–55. 20610634 10.3945/ajcn.2010.29284

[10] WHO. Serum ferritin concentrations for the assessment of iron status and iron deficiency in populations. Geneva (Switzerland): World Health Organization; 2011.

[11] Berg AH, Scherer PE Adipose tissue, inflammation, and cardiovascular disease. Circ Res 2005;96:939–49. 15890981 10.1161/01.RES.0000163635.62927.34

[12] Choi J, Joseph L, Pilote L Obesity and C-reactive protein in various populations: a systematic review and meta-analysis. Obes Rev 2013;14:232–44. 23171381 10.1111/obr.12003

[13] Wärnberg J, Marcos A Low-grade inflammation and the metabolic syndrome in children and adolescents. Curr Opin Lipidol 2008;19:11–5. 18196981 10.1097/MOL.0b013e3282f4096b

[14] Kruger HS, Pretorius R, Schutte AE Stunting, adiposity, and low-grade inflammation in African adolescents from a township high school. Nutrition 2010;26:90–9. 20005466 10.1016/j.nut.2009.10.004

[15] Hadley C, Decaro JA Testing hypothesized predictors of immune activation in Tanzanian infants and children: community, household, caretaker, and child effects. Am J Hum Biol 2014;26:523–9. 24817552 10.1002/ajhb.22558

[16] McDade TW, Rutherford JN, Adair L, Kuzawa C Population differences in associations between C-reactive protein concentration and adiposity: comparison of young adults in the Philippines and the United States. Am J Clin Nutr 2009;89:1237–45. 19225115 10.3945/ajcn.2008.27080 PMC2667466

[17] Dowd JB, Zajacova A, Aiello AE Predictors of inflammation in U.S. children aged 3-16 years. Am J Prev Med 2010;39:314–20. 20837281 10.1016/j.amepre.2010.05.014 PMC2952932

[18] Suchdev PS, Namaste SM, Aaron GJ, Raiten DJ, Brown KH, Flores-Ayala R, BRINDA Working Group. Overview of the Biomarkers Reflecting Inflammation and Determinants of Anemia project. Adv Nutr 2016;7:349–59. 26980818 10.3945/an.115.010215 PMC4785469

[19] World Bank. World development indicators [Internet].Washington (DC). 2016. [cited 2016 Apr 15]. Available from: http://data.worldbank.org/data-catalog/world-development-indicators.

[20] World Bank. Country and lending groups. Washington (DC). 2016.

[21] Erhardt JG, Estes JE, Pfeiffer CM, Biesalski HK, Craft NE Combined measurement of ferritin, soluble transferrin receptor, retinol binding protein, and C-reactive protein by an inexpensive, sensitive, and simple sandwich enzyme-linked immunosorbent assay technique. J Nutr 2004;134:3127–32. 15514286 10.1093/jn/134.11.3127

[22] WHO. Diagnosis of malaria. Geneva (Switzerland): WHO; 1990.

[23] Namaste SML, Aaron GJ, Varadhan R, Peerson JM, Suchdev PS; BRINDA Working Group. Methodologic approach for the Biomarkers Reflecting Inflammation and Nutritional Determinants of Anemia (BRINDA) project. Am J Clin Nutr 2017;106(Suppl):333S–47S. 10.3945/ajcn.116.142273 PMC5490643 28615254

[24] Thurnham D, McCabe G Influence of infection and inflammation on biomarkers of nutritional status with an emphasis on vitamin A and iron. Geneva (Switzerland): World Health Organization; 2012.

[25] Panter-Brick C, Lunn PG, Baker R, Todd A Elevated acute-phase protein in stunted Nepali children reporting low morbidity: different rural and urban profiles. Br J Nutr 2001;85:125–31. 11227041 10.1079/bjn2000225

[26] Lunn PG, Northrop-Clewes CA, Downes RM Intestinal permeability, mucosal injury, and growth faltering in Gambian infants. Lancet 1991;338:907–10. 1681266 10.1016/0140-6736(91)91772-m

[27] Keusch GT, Rosenberg IH, Denno DM, Duggan C, Guerrant RL, Lavery JV, Tarr PI, Ward HD, Black RE, Nataro JP Implications of acquired environmental enteric dysfunction for growth and stunting in infants and children living in low- and middle-income countries. Food Nutr Bull 2013;34:357–64. 24167916 10.1177/156482651303400308 PMC4643688

[28] Burke RM, Suchdev PS, Rebolledo PA, Fabiszewski de Aceituno AM, Revollo R, Iñiguez V, Klein M, Drews-Botsch C, Leon JS Predictors of pediatric inflammation in a cohort of bolivian infants and toddlers. Am J Trop Med Hyg 2016;95:954–63. 27527627 10.4269/ajtmh.16-0292 PMC5062806

[29] World Health Organization. Infant and young child feeding. Geneva (Switzerland): WHO; 2016. (Contract No.: N342.).

[30] Oluwafemi F, Ibeh IN Microbial contamination of seven major weaning foods in Nigeria. J Health Popul Nutr 2011;29:415–9. 21957681 10.3329/jhpn.v29i4.8459 PMC3190373

[31] Rohner F, Northrop-Clewes C, Tschannen AB, Bosso PE, Kouassi-Gohou V, Erhardt JG, Bui M, Zimmermann MB, Mascie-Taylor CG Prevalence and public health relevance of micronutrient deficiencies and undernutrition in pre-school children and women of reproductive age in Cote d’Ivoire, West Africa. Public Health Nutr 2014;17:2016–28. 24171836 10.1017/S136898001300222X PMC11107373

[32] Mei Z, Li H, Serdula MK, Flores-Ayala RC, Wang L, Liu JM, Grummer-Strawn LM C-reactive protein increases with gestational age during pregnancy among Chinese women. Am J Hum Biol 2016;28:574–9. 26865074 10.1002/ajhb.22837 PMC9014253

[33] Scholl TO, Chen X, Goldberg GS, Khusial PR, Stein TP Maternal diet, C-reactive protein, and the outcome of pregnancy. J Am Coll Nutr 2011;30:233–40. 21917703 10.1080/07315724.2011.10719965

